# Therapeutic Effects of Hinokitiol through Regulating the SIRT1/NOX4 against Ligature-Induced Experimental Periodontitis

**DOI:** 10.3390/antiox13050550

**Published:** 2024-04-30

**Authors:** Tae-Yeon Kim, Eun-Nam Kim, Gil-Saeng Jeong

**Affiliations:** College of Pharmacy, Chungnam National University, Daejeon 34134, Republic of Korea; taeyeon1108@o.cnu.ac.kr (T.-Y.K.); enkim@cnu.ac.kr (E.-N.K.)

**Keywords:** periodontitis, *Porphyromonas gingivalis*, hinokitiol, alveolar bone, SIRT1, NOX4

## Abstract

Hinokitiol (HKT) is one of the essential oil components found in the heartwood of Cupressaceae plants, and has been reported to have various bioactive effects, including anti-inflammatory effects. However, the improving effect of HKT on periodontitis, which is characterized by periodontal tissue inflammation and alveolar bone loss, has not been clearly revealed. Therefore, we investigated the periodontitis-alleviating effect of HKT and the related molecular mechanisms in human periodontal ligament cells. According to the study results, HKT downregulated SIRT1 and NOX4, which were increased by *Porphyromonas gingivalis* Lipopolysaccharide (PG-LPS) stimulation and were found to regulate pro-inflammatory mediators and oxidative stress through SIRT1/NOX4 signals. Additionally, by increasing the expression of osteogenic makers such as alkaline phosphatase, osteogenic induction of human periodontal ligament (HPDL) cells, which had been reduced by PG-LPS, was restored. Furthermore, we confirmed that NOX4 expression was regulated through regulation of SIRT1 expression with HKT. The in vitro effect of HKT on improving periodontitis was proven using the periodontal inflammation model, which induces periodontal inflammation using ligature, a representative in vivo model. According to in vivo results, HKT alleviated periodontal inflammation and restored damaged alveolar bone in a concentration-dependent manner in the periodontal inflammation model. Through this experiment, the positive effects of HKT on relieving periodontal tissue inflammation and recovering damaged alveolar bone, which are important treatment strategies for periodontitis, were confirmed. Therefore, these results suggest that HKT has potential in the treatment of periodontitis.

## 1. Introduction

Periodontitis is a chronic inflammatory periodontal disease prevalent worldwide, and is associated with an imbalance of oral microorganisms. The biggest characteristic of periodontitis is that it ultimately leads to tooth loss due to a decrease in periodontal tissue and alveolar bone [1]. Periodontitis mainly occurs in adults and affects approximately 20–50% of the world’s population [2,3]. Recent studies have shown that systemic diseases such as rheumatoid arthritis, cardiovascular disease, and diabetes are closely related to periodontitis [3,4,5,6]. Periodontitis begins with inflammation caused by excessive plaque in the mouth [7]. Among these, Gram-negative bacteria such as *Porphyromonas gingivalis* (*P. gingivalis*) are important pathogens in outbreaks. Lipopolysaccharide (LPS) present in their cell walls induces a strong innate immune response through the activation of pattern recognition receptors (PRRs) and Toll-like receptors (TLRs) in host cells [8,9]. This immune response produces inflammatory cytokines such as IL-6, contributing to periodontal tissue destruction [9].

Experimental animal models of periodontitis are necessary not only to confirm complex bacterial-host reactions that are difficult to confirm in cell models, but also to confirm the effects on tooth supporting tissues such as alveolar bone [10,11]. To induce periodontitis, placing ligatures around molars or administering periodontitis-causing bacteria such as *P. gingivalis* are used [10,12,13,14]. The ligature-induced model, which physically induces periodontitis by placing ligatures around the maxillary or mandibular molars, causes excessive plaque accumulation, similar to human periodontitis, and causes significant periodontal tissue destruction and alveolar bone resorption [10,12,15]. On the other hand, the oral gavage model, which administers periodontitis-causing bacteria, uses a combination of various bacterial species that contribute to causing periodontitis, such as *P. gingivalis* and *Aggregatibacter (Actinobacillus) actinomycetemcomitans* [10,12]. This method has the advantage of using specific strains to confirm the role of each strain in periodontitis [10,16,17,18]. However, compared to the oral gavage model, it was reported that the ligature-induced model induced significant periodontal inflammation and alveolar bone loss, and that this model could contribute to understanding disease pathogenesis and complex interactions between bacteria and hosts [12]. Therefore, a research model using ligation is used as a periodontitis model that can be considered in the advanced stage of periodontitis due to general plaque accumulation, and it is known as a model capable of studying not only periodontal inflammation, but also lost alveolar tissue and alveolar bone [19,20,21].

Silent information regulator 2 homolog 1 (SIRT1), which belongs to the sirtuin family, exists in the nucleus and has histone deacetylase activity dependent on nicotinamide adenine dinucleotide. Previous studies have reported that SIRT1 is involved in various pathophysiological processes, including cell growth, metabolic regulation, and aging [22,23,24]. In a study related to periodontitis, SIRT1 was reported to alleviate LPS-induced inflammation of human periodontal ligament fibroblasts (HPDLFs) through the downregulation of Toll-like receptor 4 (TLR 4) [25]. Moreover, HPDLFs stimulated with LPS suppress SIRT1 expression and simultaneously activate the NF-κB/Caspase-1 signaling pathway, causing inflammation [26]. According to research using human periodontal ligament (HPDL) cells, SIRT1 promotes osteoblastic differentiation by increasing osteogenic markers such as alkaline phosphatase (ALP) in HPDL cells [27].

NADPH oxidase 4 (NOX4) belongs to the NADPH oxidase (NOX) family and is responsible for producing reactive oxygen species (ROS) [28]. This protein is expressed in various cells, including endothelial cells and osteoclasts, and is involved in not only generating oxidative stress, but also causing excessive inflammation and promoting osteoclast differentiation [29,30]. Additionally, NOX4 levels were significantly increased in HPDLFs stimulated with *P. gingivalis* LPS (PG-LPS), which led to an increase in ROS [31]. And a recent study reported that SIRT1 reduces ROS by downregulating NOX4 [32,33,34].

Hinokitiol (HKT), also called β-Thujaplicin, is one of the essential oil components first isolated from the heartwood of Cupressaceous plants such as *Chamaecyparis taiwanensis* Masam & Suzuki and *Chamaecyparis obtusa* (Siebold & Zucc.) Endl. [35]. HKT is used as a preservative in various fields due to its antibacterial effect against a wide range of bacterial species, such as *Chlamydia trachomatis*, *Streptococcus mutans*, and *Candida albicans* [35,36,37]. In addition, HKT showed anticancer effects by inhibiting cell proliferation and inducing apoptosis in various cancer cell lines such as endometrial cancer and lung cancer [37,38,39]. Also, HKT has been reported to anti-inflammatory effects on various cell lines [40,41,42]. Additionally, HKT has various physiological effects, including antioxidant effects, neuroprotective effects, and inhibition of osteoclastogenesis [43,44,45,46]. Moreover, HKT has been reported to have anti-inflammatory effects by inhibiting the NF-κB pathway through the upregulation of SIRT1 expression in human keratinocytes [41].

Although various effects, including anti-inflammatory properties, of HKT have been revealed, studies on the effectiveness of HKT in improving periodontitis are rare. Therefore, our aim is to investigate the periodontitis-improving effect of HKT and the related intracellular signaling pathways in periodontal inflammation-induced in vitro and in vivo models.

## 2. Materials and Methods

### 2.1. Materials

Hinokitiol (CAS. 499-44-5) powder was obtained from Kwangdong Pharmaceutical Co., Ltd. (Seoul, Republic of Korea). For cell culture, Minimum Essential Medium-Alpha (α-MEM), fetal bovine serum (FBS), penicillin/streptomycin, and trypsin-ethylene diamine tetra acetic acid (EDTA) were obtained from Gibco (Grand Island, NY, USA). MTT(3-[4,5-dimethylthiazol-2-yl]-2,5-diphenyl tetrazolium bromide) was purchased from Amresco Inc (Cleveland, OH, USA). *P. gingivalis* Lipopolysaccharide (PG-LPS) was obtained from Invivo Gen (San Diego, CA, USA). Tumor necrosis factor-α (TNF-α) and IL-6, IL-1β ELISA assay kit was also obtained from a R&D system (Minneapolis, MN, USA). For Western blotting, anti-SIRT1 (120 kDa), SOD (18 kDa), CAT (60 kDa), GPx (22 kDa), iNOS (130 kDa), COX-2 (74 kDa), and β-actin (45 kDa) antibodies were purchased from Cell Signaling Technology Inc. (Danvers, MA, USA), and anti-NOX4 (67 kDa) was obtained from Novus Biologicals (Littleton, CO, USA). Secondary antibodies were purchased from Santa Cruz Biotechnology Inc (Dallas, TX, USA). A Hybond ECL PVDF was purchased from Amersham Pharmacia Biotech Inc. (Piscataway, NJ, USA). To detect Western blotting, a Western blotting detection system was provided by Advansta Inc. (Santa Clara, CA, USA). For osteoblast staining and ROS production assay analysis, Ailzarin Red S and Dichlorofluorescin diacetate (DCF-DA) was obtained from Sigma-Aldrich (St. Louis, MO, USA). An EX-527 as SIRT1 inhibitor was obtained from Sigma-Aldrich (St. Louis, MO, USA).

### 2.2. Cell Culture

The isolation and culture of human periodontal ligament (HPDL) cells was approved by the Kyungpook National University Institutional Review Board (Daegu, Republic of Korea) (KNU 2017-78) and was provided by Professor Jae-Young Kim, College of Dentistry, Kyungpook National University. Briefly, HPDL cells were obtained from each donor’s third molar, and the PDL was gently separated from the root surface and incubated with 3 mg/mL collagenase type I (Worthington Biochem, Freehold, NJ, USA) and 4 mg/mL dispase (Roche, Mannheim, Germany) solution for 1 h. PDL samples from different individuals were pooled to obtain a single-cell suspension by passing the cells through a 70 m strainer (Falcon, BD Labware, Franklin Lakes, NJ, USA), and then single-cell suspensions (1 × 10^4^ cells) were spread in 10 cm cultures. Afterwards, the culture was cultured in α-MEM supplemented with 10% (*v*/*v*) fetal bovine serum (FBS) and 1% penicillin/streptomycin (Gibco BRL, Grand Island, NY, USA), and the cells were maintained at 37 °C in 5% CO_2_ [47].

### 2.3. Cell Viability of HKT

For the determination of cell viability, HPDL cells (5 × 10^3^ cells/well) were seeded and incubated in a 96-well plate for 24 h (37 °C, 5% CO_2_) to measure the cell viability of HKT. Then, HPDL cells were treated for 48 h at all concentrations of HKT (0.25 μM, 0.5 μM, 1 μM, 2 μM). An amount of 5 mg/mL MTT(3-[4,5-dimethylthiazol-2-yl]-2,5-diphenyl tetrazolium bromide) was added to 50 μL of cell suspension, followed by incubation for 4 h. The medium was removed and added 100 μL dimethyl sulfoxide (DMSO). Absorbance of dissolved formazan crystals was measured with a microplate reader (Tecan Trading AG) (Männedorf, Switzerland) at 540 nm. To determine the cell proliferation, HPDL cells (5 × 10^3^ cells/well) were seeded in a 24-well plate and treated with HKT (0.25 μM, 0.5 μM, 1 μM, 2 μM) for 48 h. Incucyte^®^ Live-Cell analysis systems (Göttingen, Germany) were used for cell counting. For the cell-count analysis calculation, the cell counts of the stained control group were converted into percentages, and the counts of the HKT treated group were averaged.

### 2.4. Evaluation of Inhibition of ROS Generation by HKT

To determine the level of intracellular ROS, HPDL cells were incubated in a 6-well plate for 24 h, and then treated with all concentrations of HKT (0.25 μM, 0.5 μM, 1 μM, 2 μM) and 10 μg/mL PG-LPS and incubated in humidified conditions (37 °C, 5% CO_2_) for 2 h. After incubation, the medium with 10 μM DCF-DA was treated to each well and incubated in humidified conditions (37 °C, 5% CO_2_). The cells were washed with PBS, treated with 1% trypsin-EDTA solution, and harvested. Then, they were washed with PBS, treated again, and incubated for 30 min. At same time, the amount of change in ROS was analyzed using the Incucyte^®^ Live-Cell analysis system.

### 2.5. RT-qPCR

A real-time PCR analysis was performed to measure target mRNA gene levels. Total RNA of HPDL cells treated HKT was extracted with TRIzol/chloroform reagent (Bioneer, Daejeon, Republic of Korea) and reverse-transcribed into cDNA using a TOPscriptTM RT DryMix (dT18) kit (enzynomics, Daejeon, Republic of Korea) according to the instructions. To amplify the cDNA, a RT-qPCR was performed with TB Green^®^ Premix Ex TaqTM II (Tli RNaseH Plus) (TaKaRa, Tokyo, Japan). Thermal cycling conditions were 1 cycle at 95 °C initial denaturation for 30 s and 45 cycles at 95 °C denaturation for 5 s, and 60 °C annealing for 34 s. The target gene expression levels were normalized to *β-actin* or *gapdh* gene, which are housekeeping genes. The primers used in this study were synthesized with a Bioneer Co. (Daejeon, Republic of Korea). The primer pairs sequences are listed in Table 1.

### 2.6. Western Blotting Analysis

Total protein levels in HPDL cells were determined by immunoblotting. The HPDL cells were washed by PBS followed, then after, lysed using RIPA (Radio-Immunoprecipitation Assay) buffer for 30 min. And lysates were centrifuged at 12,000 rpm for 15 min. Afterwards, Bradford (Sigma Alrich, St. Louis, MO, USA) analysis was performed to quantify protein was performed. Then, the equal amount of protein (20 μg) was separated by 12% SDS-PAGE and transferred to PVDF membranes. The membranes were blocked in 5% (*w*/*v*) skim milk dissolved in TBS-T buffer (10 mM Tris (pH 8.0) and 150 mM NaCl) at room temperature for 1 h. After blocking, the membranes were rinsed with TBST buffer for 1 h and incubated with primary antibody overnight at 4 °C. The membranes incubated the primary antibody were washed with Tris Buffered Saline with Tween 20 (TBS-T) buffer and then incubated in a secondary antibody. The protein bands were detected with Enhanced Chemiluminescence (ECL) Western blotting detection reagents (Thermo Fisher Scientific; Waltham, MA, USA). The band images were scanned with an ChemiDoc XRS+ system (Bio-Rad, CA, USA). The detected bands were quantified using the image J 1.54i version software (National Institutes of Health, Bethesda, MD, USA).

### 2.7. Mineralization Assay

For osteogenic induction, HPDL cells were seeded in a 24-well plate at 5 × 10^3^ cells/well and cultured in osteo-induction medium (OIM) containing 50 μg/mL ascorbic acid and 10 mM β-glycerophosphate for 7 days. Once the mineralized nodules were formed, they were fixed with 4% polyformaldehyde. After 30 min, fixed nodules were stained at pH 4.3 with 2% Alizarin Red S for 30 min at room temperature and then washed with deionized water. A microscope (Nikon, Japan) was used to observe the staining result. To quantify the amount of calcium deposition, it was dissolved in 10% CPC (cetylpyridinium chloride) and the absorbance was measured at 560 nm using a multifunctional microplate reader (M1000 Pro, TECAN, Männedorf, Switzerland) [48].

### 2.8. Animals

All animal experiments were conducted according to the “Guidelines in Use of Animal” established by the Chungnam National University Institutional Animal Care and Use Committee (Daejeon, Republic of Korea). And the experimental procedure was approved by the Chungnam National University Institutional Animal Care and Use Committee (202212-CNU-259). Male 8-week-old Sprague–Dawley rats were purchased from Samtako Inc. (Osan, Republic of Korea). Experiments with rats were conducted in the animal laboratory of College of Chungnam National University (Daejeon, Republic of Korea).

### 2.9. Ligature-Induced Periodontal Inflammation Model

Eight-week-old male Sprague Dawley rats (*n* = 4, in each group, total 24 rats, each group: control group, only ligation induced group, ligation+HKT 5 μm, ligation+HKT 10 μM, ligation+HKT 20 μM, ligation+HKT 40 μM) were anesthetized by CO_2_ inhalation, and their right maxillary first molars were fixed and then ligated by knotting with non-absorbable silk. The ligation site was checked every day to determine whether periodontal inflammation was induced, and a free diet was administered. Afterwards, on the sixth day of induction, the non-wettable silk ligated to the first molar was removed, and the first molar was also extracted. The formation of a tooth pocket in the area of the extracted first molar was confirmed, and bleeding was stopped through sufficient pressure. Then, after, 5, 10, 20, and 40 μM of HKT was applied to the extracted tooth pocket, the upper part of the periodontal pocket was sealed with Pluronic^®^ F-127 (Sigma-Aldrich, St. Louis, MO, USA) to be released HKT for 9 days. After the experiment was completed, the rat was sacrificed, and the upper and lower jaws were separated from the skull. Pluronic^®^ F-127 was removed from the upper-right first molar and washed with distilled water. After de-fleshing, hemi-maxillae were stained with 1% methylene blue (CAS: 7220-79-3, Sigma-Aldrich, St. Louis, MO, USA) for 1 min, and the stained maxillae were washed with distilled water, the images of bone loss and alveolar bone were captured using 2D/3D-micro-CT by staining the methylene blue. The overall experimental sequence is summarized in Figure below.

### 2.10. Micro-Computed Tomography (Micro-CT) Imaging and Analysis

Micro-CT (Quantum FX micro-CT, Perkin Elmer, Waltham, MA, USA) were scanned at tube current (160 uA), tube voltage (90 kVp), imaging time (180 s), Pixil size (10 μM), and FOV (field of view, 5 mm). To measure bone mineral density (BMD), the direction was changed to a coronal section. The raw data obtained from Micro-CT were loaded into CTAn, and the images were scanned. BMD was determined based on the above thresholds. The region of interest (ROI) photographed for measuring bone density was set as the area for the alveolar bone formed in the extracted area where the teeth were located. Data were presented as the mean ± SD. The significance of the data was analyzed by one-way ANOVA using SPSS Statistics (Armonk, NY, USA).

### 2.11. Histological Staining

Hematoxylin and eosin (H&E) staining was performed to confirm inflammatory infiltration of periodontal tissue from the extracted maxillary skull. Briefly, the entire maxillary skull extracted was stored in 10% formalin and then embedded in paraffin. Afterwards, it was cut into 5 μm pieces and fixed on a slide. The tissue fixed on the slide was stained with H&E, and the degree of periodontal tissue invasion of the fixed slide tissue was observed using a fluorescence Olympus IX microscope 71-F3 2PH (Tokyo, Japan), and the same region of interest was set for the periodontal extraction site of all samples to create a comparison for the ligature-induced group. The area of the white soft tissue was expressed as a percentage compared to the control group.

### 2.12. Statistical Analysis

All experiments in this study were performed independently at least three times, and data were described as the mean ± standard deviation (SD). Prism 9.0.0 (GraphPad Software, San Diego, CA, USA) was used for statistical analysis of data, and one-way ANOVA was used to analyze significance (*p*-value); *p* < 0.05 was considered statistically significant.

## 3. Results

### 3.1. HKT Is Not Cytotoxic in HPDL Cell

**Figure 1:**
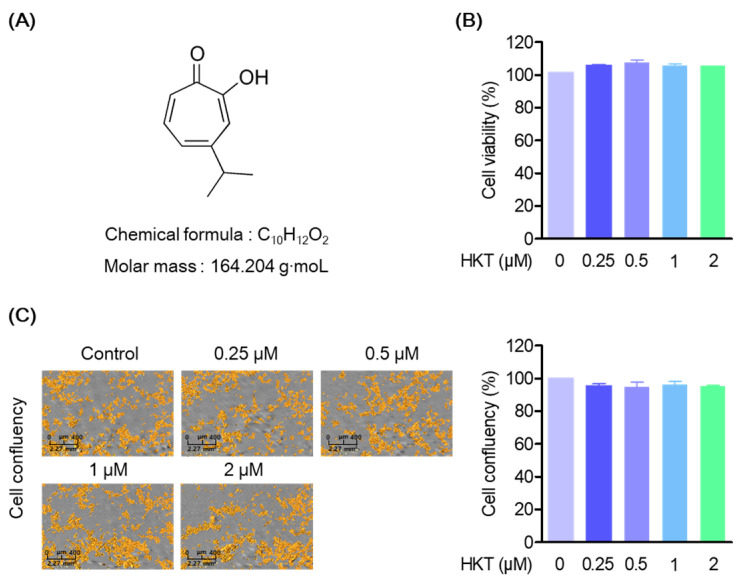
HKT structure and cell viability to HPDL cells. (**A**) HKT structure. (**B**) MTT assays results. An MTT assay was performed to measure the cell viability of HKT at the indicated concentrations (0.25, 0.5, 1, or 2 µM). (**C**) Cell coefficient assay (scale bar 2.27 mm^2^) results. A cell coefficient assay was performed to determine whether the indicated concentrations of HKT inhibited cell proliferation. Scale bar: 400 μm, 2.27 mm^2^.

HKT has the structure of a monoterpene (Figure 1A), and in this study, the cytotoxicity of HKT was first evaluated in HPDL cells to identify appropriate test concentrations. We also evaluated the effects of HKT on cytotoxicity, cell morphological changes, or proliferation in HPDL cells. First, HPDL cells were cultured and treated with HKT at 0, 0.25, 0.5, 1, or 2 μM for 48 h, and an MTT analysis was performed. In the MTT analysis results, HKT showed a similar amount of formazan formation as the control group, and the actual absorbance measurement results did not show significant toxicity (Figure 1B). In addition, as a result of the analysis using live-cell systems to evaluate the effect of HKT on morphological changes or proliferation of cells along with cell toxicity evaluation, it was confirmed that HKT did not affect the morphological changes and proliferation of HPDL cells within the indicated concentration (Figure 1C).

### 3.2. HKT Inhibits Oxidative Stress Generated by PG-LPS

**Figure 2:**
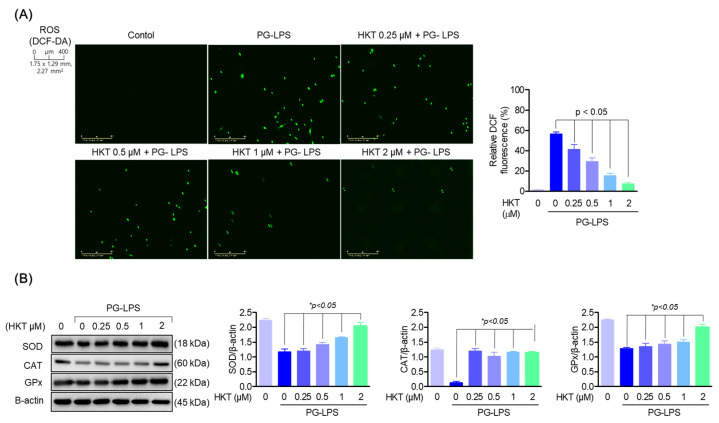
HKT reduces the oxidative stress caused by PG-LPS. (**A**) DCF-DA was performed to determine whether the indicated concentrations of HKT inhibit ROS production. (**B**) Immunoblotting was performed to confirm the regulation of protein expression of SOD (18 kDa), CAT (60 kDa), and GPx (22 kDa) by the indicated concentrations of HKT (0.25, 0.5, 1, or 2 µM). Results are expressed as the mean ± SEM of three independent experiments (* *p* < 0.05). Scale bar: 400 μm, 1.75 × 1.29 mm, 2.27 mm^2^.

ROS is a factor that can cause periodontitis, occurs during periodontitis, and acts to exacerbate periodontal disease [7]. Therefore, antioxidant effects on ROS are important in the treatment of periodontitis. Therefore, we evaluated the effects of HKT on ROS and antioxidant enzymes generated in HPDL cells stimulated with PG-LPS. As can be seen from the research results, it was confirmed that HKT downregulates the level of ROS increased by PG-LPS in a concentration-dependent manner (Figure 2A). In addition, we were able to confirm the results of restoring the protein expression level of antioxidant enzymes such as superoxide dismutase (SOD), catalase (CAT), and glutathione peroxidase (GPx) lost by PG-LPS (Figure 2B). The inhibition of HKT’s increased ROS production in PG-LPS-stimulated HPDL cells, and its effect in controlling the recovery of lost antioxidant enzyme proteins is a result that shows the potential for alleviating periodontitis.

### 3.3. HKT Restores the Induction of Osteoblast Differentiation Suppressed by PG-LPS.

**Figure 3:**
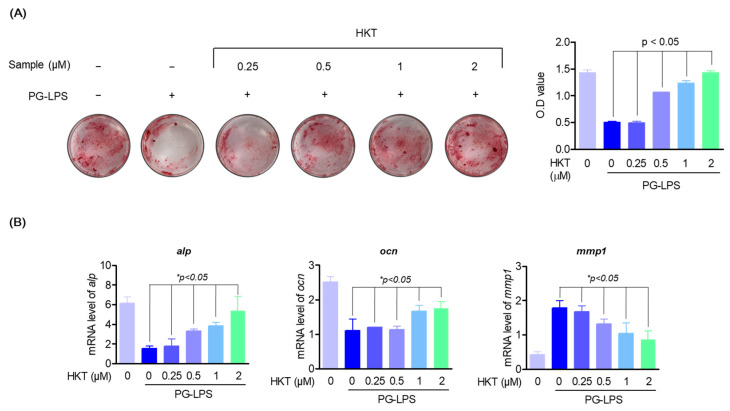
HKT restores the induction of osteoblast differentiation suppressed by PG-LPS. (**A**) Alizarin Red S staining (24 well full hole) was performed to confirm whether the indicated concentration of HKT induced mineralization in HPDL cells. (**B**) RT-qPCR was performed to determine the mRNA expression levels of bone formation markers *alp*, *ocn*, and *mmp-1*, one of the factors promoting bone resorption, at the indicated HKT concentrations (0.25, 0.5, 1, or 2 µM). Results are expressed as the mean ± SEM of three independent experiments (* *p* < 0.05).

Deficiency of periodontal ligament cells due to periodontitis can weaken periodontal connective tissue and inhibit the formation of osteoblasts, and the differentiation of periodontal ligament cells into osteoblasts, along with suppression of periodontal inflammation, has been known to be an important therapeutic strategy in the treatment of periodontitis [49]. Therefore, to confirm the effect of HKT on osteoblast differentiation of HPDL cells, osteoblast differentiation was induced by replacing media with α-MEM containing 50 μg/mL ascorbate and 10 mM β-glycerophosphate for 7 days. As a result, it was con-firmed that the differentiation of HPDL cells into an osteoblast suppressed by PG-LPS (10 μg/mL) was increased again by HKT (Figure 3A). In addition, to determine whether HKT inhibits the gene expression of osteoblast markers such as alkaline phosphatase (ALP) and osteocalcin (OCN) and matrix metalloproteinase (MMP) such as MMP-1 in early osteoblast differentiation, a RT-qPCR was performed. As a result, HKT increased the expression levels of *alp* and *ocn*, which were lost by PG-LPS, and reduced the expression of protease *mmp-1*, which was increased by PG-LPS (Figure 3B). Therefore, it was confirmed that HKT restores the osteoblast differentiation-inducing specific markers suppressed by PG-LPS during the osteoblast differentiation induction process of HPDL cells, and has a protease inhibitory effect.

### 3.4. HKT Downregulate PG-LPS-Induced Expression of Pro-Inflammatory Cytokines and Mediators in HPDL Cells

**Figure 4:**
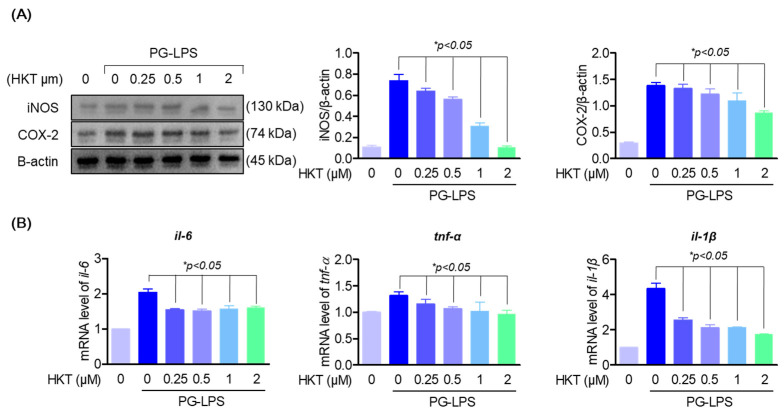
HKT downregulate PG-LPS induced expression of pro-inflammatory cytokines and mediators in HPDL cells. (**A**) Western blotting was performed to determine whether the indicated concentrations of HKT (0.25, 0.5, 1, or 2 µM) modulate the increased iNOS (130 kDa) and COX-2 (74 kDa) expression caused by PG-LPS. (**B**) RT-qPCR was performed to determine whether the expression of *il-6, il-1β*, and *tnf-α* was regulated by the indicated concentrations of HKT (0.25, 0.5, 1, or 2 µM). Results are expressed as the mean ± SEM of three independent experiments (* *p* < 0.05).

It was thought that the oxidative stress inhibitory effect of HKT shown previously could have a direct alleviating effect on periodontitis. Therefore, we evaluated the effect of HKT on pro-inflammatory mediators iNOS and COX-2 and the major inflammatory cytokines of periodontitis, IL-6, IL-1β, and TNF-α, in an actual inflammatory situation. First, HKT downregulated the protein expression of proinflammatory mediators iNOS and COX-2, which were increased with PG-LPS stimulation, in a concentration-dependent manner (Figure 4A). Likewise, the increased levels of pro-inflammatory cytokines *il-6*, *il-1β*, and *tnf-α* genes were downregulated (Figure 4B). These results demonstrate the anti-inflammatory effect of HKT, and in particular, the downregulation effect of major pro-inflammatory cytokines in periodontitis suggests the possibility that HKT may have a periodontitis control effect in actual periodontitis situations.

### 3.5. HKT Regulates SIRT1/NOX4 Protein Expression

**Figure 5:**
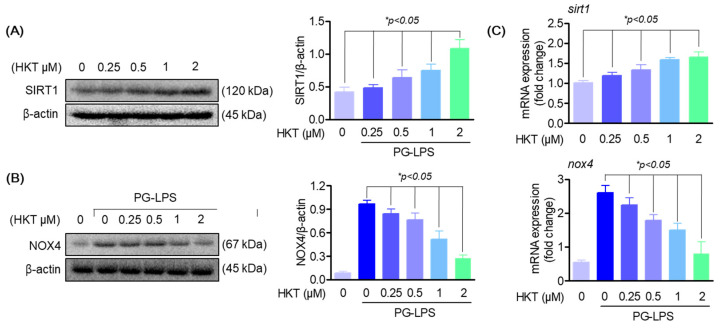
HKT regulates SIRT1/NOX4 expression. (**A**) HKT increases SIRT1 (120 kDa) expression. Results of confirming the protein expression levels of SIRT1. Immunoblotting was performed to determine the level of SIRT1 expression in HPDL cells. HKT at the indicated concentrations (0.25, 0.5, 1, or 2 µM) increased SIRT1 expression. (**B**) HKT inhibits NOX4 (67 kDa) expression in HPDL cells. At the indicated concentrations (0.25, 0.5, 1, or 2 µM), (**C**) mRNA expression levels of SIRT1 and NOX4. Similar to the protein expression pattern, HKT increases the expression of SIRT1, while suppressing the expression of NOX4. Results are expressed as mean ± SEM of three independent experiments (* *p* < 0.05).

SIRT1 expression has been reported to reduce ROS production by downregulating NOX4 in several cell models, and is also known to regulate inflammatory responses through downregulating Toll-like receptor 4 (TLR 4) [25,32,33,34]. Therefore, to determine whether HKT exerts antioxidant effects through the SIRT1/NOX4 pathway in HPDL cells, we first determined whether SIRT1 and NOX4 are regulated by HKT. First, the effect of HKT treatment on the SIRT1/NOX4 pathway was explored through a Western blotting assay, and the results showed that HKT treatment upregulated the protein expression of SIRT1/NOX4 in a concentration-dependent manner (Figure 5A,B). Therefore, we suggest the possibility that the anti-inflammatory, antioxidant, and osteoblast differentiation recovery effects against PG-LPS in HPDL cells shown previously may be due to the effect of HKT inducing SIRT1/NOX4 protein expression.

### 3.6. HKT Alleviates ROS Production by Regulating the SIRT1/NOX4 Pathway

**Figure 6:**
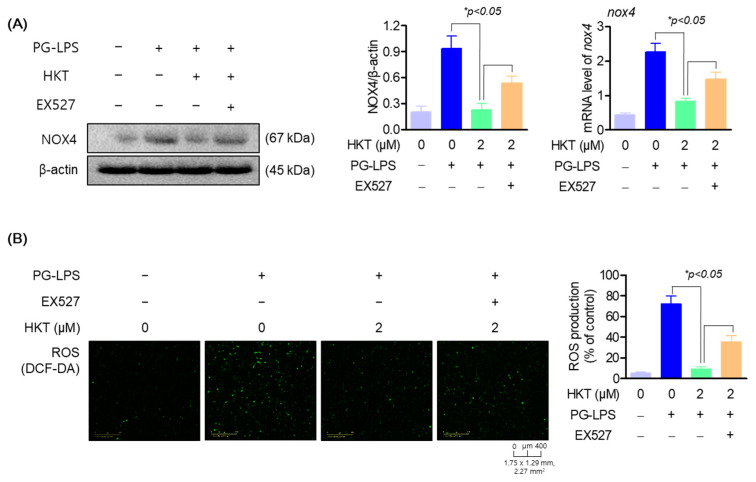
HKT reduces ROS production by regulating the SIRT1/NOX4 pathway. (**A**) HKT downregulates NOX4 (67 kDa), which was increased with PG-LPS by increasing SIRT1 expression. (**B**) HKT downregulates ROS production through regulation of the SIRT1/NOX4 pathway. Results are expressed as mean ± SEM of three independent experiments (* *p* < 0.05). Scale bar: 400 μm, 1.75 × 1.29 mm, 2.27 mm^2^.

As shown in Figure 5, HKT upregulated the protein expression of SIRT1/NOX4 in HPDL cells. Therefore, to confirm the effect of upregulation of SIRT1/NOX4 protein expression in HKT on oxidative stress induced by PG-LPS, changes in NOX4 expression were evaluated after inhibiting SIRT1 by treating EX-527, a specific inhibitor of SIRT1. As a result of the study, in the group treated with PG-LPS, the protein expression level of NOX4 increased as before, but it was confirmed to be downregulated with HKT treatment; and the results of pretreatment with EX-527, a SIRT1 inhibitor, showed that regarding the NOX4 protein of HKT, it was found that the expression down-regulation effect was reversed (Figure 6A). In addition, the level of ROS generated by PG-LPS was also downregulated with HKT treatment, but this effect was confirmed to be reversed with EX-527 treatment (Figure 6B). These results indicate that the downregulation of NOX4 through SIRT1 regulation is directly related to the antioxidant effect of HKT shown previously, and suggest that HKT, as a SIRT1 regulator, has the potential to alleviate periodontal inflammation caused by ROS accumulation.

### 3.7. HKT Regulates Pro-Inflammatory Cytokines and Osteoblast Differentiation-Inducing Specific Genes through the SIRT1/NOX4 Pathway

**Figure 7:**
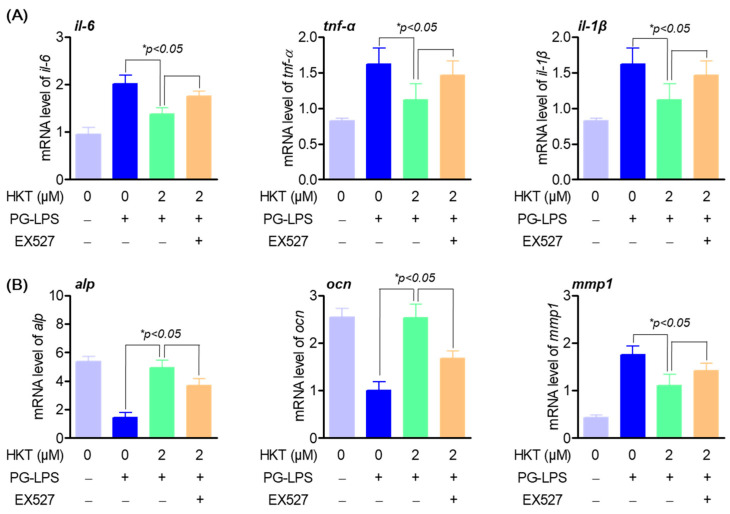
HKT promotes anti-inflammatory and osteogenic induction through regulating SIRT1/NOX4 pathway. (**A**) Anti-inflammation effects of HKT through SIRT1/NOX4 regulation. HKT reduces the expression of pro-inflammatory cytokines such as IL-6, which was increased by PG-LPS through regulating the SIRT1/NOX4 pathway. (**B**) HKT restores the reduced osteogenic induction caused by PG-LPS by regulating the SIRT1/NOX4 pathway. Results are expressed as mean ± SEM of three independent experiments (* *p* < 0.05).

As already known, the regulation of oxidative stress through SIRT1/NOX4 regulation plays a very important role not only in inflammation, but also in the bone formation process [7]. Therefore, we evaluated the effect of treatment with EX527, an inhibitor of SIRT1, on the previously shown anti-inflammatory effect on PG-LPS of HKT and on the osteoblastic differentiation process of HPDL cells. Previous research results showed that HKT reduced the gene level of pro-inflammatory cytokines such as *il-6*, *tnf-α*, and *il-1β*, which were increased by PG-LPS, but this effect was suppressed with EX-527 treatment (Figure 7A). In addition, HKT restored gene expression of early osteoblast differentiation-specific markers *alp* and *ocn*, which were suppressed with PG-LPS treatment, but this effect was reversed with EX-527 treatment. It was confirmed that the effect of suppressing expression of the protease *mmp1* gene was also reversed (Figure 7B). Therefore, the results of this study show that HKT not only has an antioxidant effect through regulation of SIRT/NOX4, but also plays a role in restoring anti-inflammatory and lost osteoblast differentiation ability of HPDL cells. Ultimately, this suggests that it plays an important role in suppressing periodontal inflammation, which is the main treatment strategy for periodontitis, and in protecting and recovering periodontal tissue.

### 3.8. HKT Recovers Damaged Alveolar Bone in a Periodontal Inflammation Model

**Figure 8:**
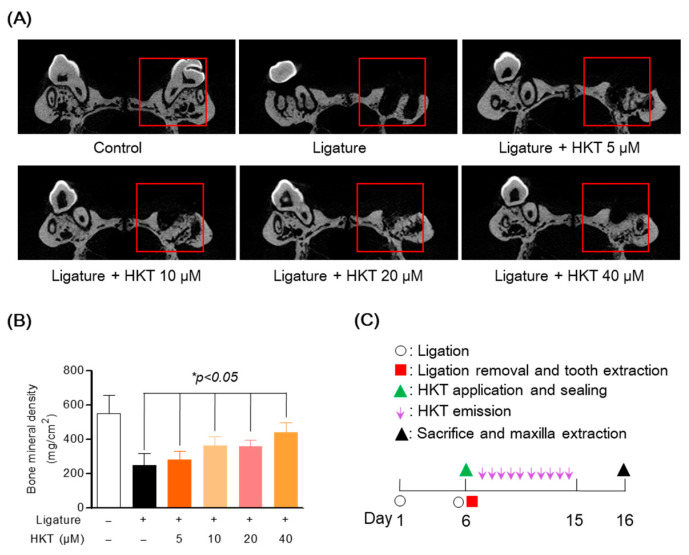
Alveolar bone recovery effect of HKT in periodontal inflammation model. (**A**) Micro-CT analysis of a maxilla with periodontitis. Alveolar bone damaged by ligature was recovered by HKT in a concentration-dependent manner. (**B**) Confirmation of bone density recovery effect by HKT in extracted socket. (**C**) Summary of the entire experimental schedule. Analysis tables were determined using CTAn software 1.18 version (Bruker, Cambridge, UK). Results are expressed as mean ± SEM of five rats (* *p* < 0.05). (Red box: Analysis region of interest (ROI)).

In order to provide more solid evidence for the effect of HKT on suppressing periodontitis revealed in previous in vitro studies, the effect of HKT was evaluated using a rat model in which periodontal inflammation was induced using a ligature commonly used in periodontitis research. After the study was completed, the recovery effect of damaged alveolar bone was confirmed using micro-CT of the maxilla from which the molars were extracted. To induce periodontitis, the maxillary first molars were ligated with silk for 6 days, the first molars were extracted, and HKT was applied for 9 days. The schedule of the entire study is summarized in Figure 8C. In the research results, it was confirmed that periodontal tissue damaged by ligature was recovered in a concentration-dependent manner by HKT application, and it was confirmed through BMD analysis that new periodontal tissue and alveolar bone were formed in red box, the pocket of the extracted tooth area (Figure 8A,B). These results indicate that HKT directly restores damaged periodontal tissue due to the induction of periodontal inflammation, and are evidence that supports the claims of the in vitro study results shown earlier.

### 3.9. HKT Exhibits Periodontal Tissue Recovery and Anti-Inflammatory Effects in a Periodontal Inflammation Model

**Figure 9:**
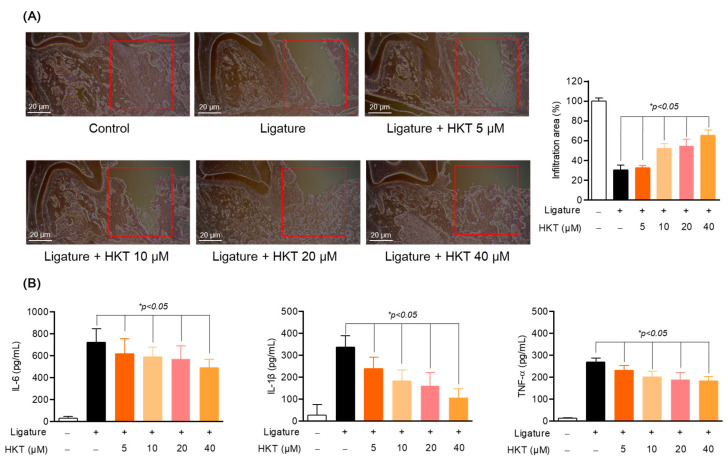
HKT restores periodontal tissue in a concentration-dependent manner and simultaneously regulates systemic inflammation. (**A**) H&E staining of periodontal tissue. (B) mRNA expression of IL-6, TNF-α, and IL-1β in serum. Results are expressed as mean ± SEM of five rats (* *p* < 0.05). (Red box: Analysis region of interest (ROI)). Scale bar: 20 μm.

In addition to the protection and recovery effect of periodontal tissue from ligature-induced periodontal inflammation, actual suppression of periodontal inflammation can be said to be a fundamental strategy for periodontitis treatment. Therefore, we sought to evaluate the direct effect of HKT on periodontal inflammation. Therefore, to evaluate the periodontal regeneration and anti-inflammatory effects of HKT, periodontal tissue and serum were harvested from an in vivo model with or without inducing periodontitis. Firstly, the effect of HKT on inflammatory cell infiltration caused by periodontitis was confirmed through H&E staining. As can be seen in Figure 9A, the induction of ligature did not regenerate new periodontal tissue from the extracted tooth area, but it was confirmed that new periodontal tissue formed in white was regenerated by treatment with HKT. In addition, as a result of evaluating the infiltration area by setting a certain area of interest from the area where the tooth was extracted, it was confirmed that the result was recovered with HKT treatment (Figure 9A). In addition, as a result of confirming the systemic inflammation levels using serum, the levels of pro-inflammatory cytokines IL-6, TNF-α, and IL-1β were decreased in a concentration-dependent manner by HKT (Figure 9B). Therefore, it was determined that HKT can not only restore periodontal tissue damaged by inflammation, but also regulate systemic inflammation.

## 4. Discussion

During the periodontal inflammation process, inflammation of the periodontal tissue leads to loss of alveolar bone, ultimately resulting in tooth loss. In the process of developing periodontitis, LPS, a component of Gram-negative bacteria, reacts with Toll-like receptor 4 (TLR 4), a membrane protein of host cells, and activates inflammatory pathways such as NF-ĸB into cells [9,50,51]. Because this increases the secretion of inflammatory cytokines, if it is excessive or continues for a long period of time, and it can cause chronic inflammation or autoimmune reactions. It can also accelerate periodontal tissue destruction and alveolar bone loss by activating osteoclasts and collagenase [7]. Therefore, in order to improve periodontitis, it is necessary to suppress an excessive inflammatory response and restore damaged alveolar bone.

In periodontal ligament tissue, which is one of the important tissues in the treatment of periodontitis, periodontal ligament (PDL) cells perform various roles such as tooth support, nutrient supply, and tissue regeneration, and can differentiate into osteoblasts or periodontal ligament fibroblasts [52]. Due to these abilities, these cells are important for periodontal tissue regeneration and alveolar bone recovery, and considering that these effects are an important strategy for improving periodontitis, experiments with PDL cells are necessary in periodontitis research.

HKT is a component with several biological activities extracted from the heartwood of cupressaceous plants [35]. Among these activities, there were studies related to periodontitis. The study results showed that HKT suppressed inflammation and alveolar bone loss caused by periodontitis; However, this study did not confirm the effect of HKT on cell lines other than oral periodontal ligament-related cells, such as human gingival fibroblasts [52].

Our results showed that HKT reduced the expression of proinflammatory cytokines and inflammatory mediators, such as IL-6, in HPDL cells. Additionally, the expression level of MMP-1, which contributes to alveolar bone loss, decreased, whereas the expression of osteogenic markers increased. And while ROS, a factor that causes and worsens periodontitis, decreased in a concentration-dependent manner, the expression of antioxidant enzymes increased.

It was confirmed that this antioxidant effect occurs through HKT regulating the SIRT1/NOX4 pathway. The sirtuin family, including SIRT1, regulates cellular stress, inflammation, aging, and cell death by inducing deacetylation of various target proteins such as intracellular Forkhead-box protein O3a (FOXO3a) and SOD [53]. Among these, SIRT1 has been confirmed to be involved in various intracellular pathways such as antioxidant, anti-inflammatory, and osteoblast differentiation [22,23,24,25,26,27]. According to recent studies, SIRT1 was reported to reduce oxidative stress by regulating NOX4 expression [19,20,21]. In particular, SIRT1/NOX4 regulation is considered important in regulating oxidative stress in periodontitis, based on the fact that the interaction between NOX4 and the redox system is important for ROS formation that accelerates periodontitis [7,31]. Therefore, we hypothesized and proved that HKT would exert antioxidant effects by regulating the SIRT1/NOX4 pathway. In addition to the antioxidant effect, we confirmed that HKT has an important inflammation-alleviating effect and osteogenic induction-promoting effect in improving periodontitis through the SIRT1/NOX4 pathway.

Based on these in vitro results, the effect of HKT on improving periodontitis was confirmed using the ligation-induced periodontitis model, which is a representative in vivo model of periodontitis. According to the results, HKT reduced inflammatory infiltration of periodontal tissue caused by ligation in a concentration-dependent manner, and also restored damaged alveolar bone. At the same time, serum analysis showed decreased levels of pro-inflammatory cytokines.

*P. gingivalis*, a gram-negative anaerobic bacterium, is the main pathogen of periodontitis. It is closely related to the destruction of periodontal tissue through its involvement in proteolytic enzymes and the formation of plaque, which causes periodontitis. As a causative bacteria of periodontitis, it is known to play a role in increasing pathogenicity in various stages from the initial stage of periodontitis to rapidly progressive periodontitis [54,55]. In addition to these effects, considering that oral gram-negative bacteria are involved in the cause of periodontitis, in addition to the effects of alleviating periodontal tissue inflammation and restoring damaged alveolar bone, the antibacterial effect against the typical causative bacteria of periodontitis is also a treatment strategy for periodontitis [7]. Previously, HKT was known to exhibit anti-inflammatory effects and various physiological activities [40,41,42,43,44,45,46], but on the other hand, high-dose oral administration in rats is also known to cause developmental toxicity in pregnant rats [56]. These result of chronic toxicity that may occur due to long-term, high-concentration oral administration, the doses (5, 10, 20, 40 µM) used in this study were already much lower than the toxic dose, and were shown to be effective in treating periodontal inflammation by applying it to the periodontal tissue rather than by oral administration. However, the toxic effects of HKT on periodontal tissue have not yet been revealed, and this study has limitations regarding the potential side effects and toxicity of HKT.

The results of this study showed that HKT alleviated inflammation and restored damaged alveolar bone in both in vitro and in vivo models. Therefore, it is considered that HKT has the potential to improve periodontitis by restoring damaged periodontal tissue and alveolar bone due to excessive inflammation.

## 5. Conclusions

In this study, whether HKT has a therapeutic effect on periodontitis was confirmed in in vitro and in vivo models. In HPDL cells stimulated with PG-LPS, HKT decreased the expression of pro-inflammatory cytokines and MMP-1, while it increased the expression of markers related to osteogenesis. Furthermore, HKT reversed the increased ROS and decreased antioxidant enzyme expression caused by PG-LPS. In the mechanism study, it was confirmed that these antioxidant and anti-inflammatory effects of HKT occurred due to the regulation of the SIRT1/NOX4 pathway. Similar to the in vitro results, in the ligature-induced periodontal inflammation model, periodontal tissue inflammation, and damaged alveolar bone were recovered with HKT. Therefore, this suggests that HKT has the potential to improve periodontitis through the antibacterial effect and the recovery of periodontal tissue inflammation and damaged alveolar bone.

## Figures and Tables

**Table 1:** Primer sequences.

Target Gene	Sequence (5′→3′)		Accession Number
*il-6*	Forward	AGTGAGGAACAAGCCAGAGC	NM_000600.4
	Reverse	GTCAGGGGTGGTTATTGCAT	
*il-1β*	Forward	AACCTCTTCGAGGCACAAGG	NM_000576.2
	Reverse	GTCCTGGAAGGAGCACTTCAT	
*tnf-α*	Forward	GCCTCTTCTCCTTCCTGATCGT	NM_000594.2
	Reverse	TGAGGGTTTGCTACAACATGGG	
*alp*	Forward	TGCAGTACGAGCTGAACAGG	NM_000478
	Reverse	GTCAATTCTGCCTCCTTCCA	
*ocn*	Forward	CGCTACCTGTATCAATGGCTGG	NM_199173
	Reverse	CTCCTGAAAGCCGATGTGGTCA	
*mmp-1*	Forward	ATGAAGCAGCCCAGATGTGGAG	NM_002421
	Reverse	TGGTCCACATCTGCTCTTGGCA	
*sirt1*	Forward	TAGACACGCTGGAACAGGTTGC	NM_012238.5
	Reverse	CTCCTCGTACAGCTTCACAGTC	
*nox4*	Forward	GCCAGAGTATCACTACCTCCAC	NM_016931
	Reverse	CTCGGAGGTAAGCCAAGAGTGT	
*β* *-actin*	Forward	AGAGCTACGAGCTGCCTGAC	NM_001101
	Reverse	AGCACTGTGTTGGCGTACAG	
*gapdh*	Forward	TGTTCGTCATGGGTGTGAAC	NM_002046
	Reverse	GTCTTCTGGGTGGCAGTGAT	

## Data Availability

All data generated or analyzed during this study are included in this published article.
